# Synthesis and Complete Antimicrobial Characterization of CEOBACTER, an Ag-Based Nanocomposite

**DOI:** 10.1371/journal.pone.0166205

**Published:** 2016-11-08

**Authors:** O. E. Jaime-Acuña, A. Meza-Villezcas, M. Vasquez-Peña, O. Raymond-Herrera, H. Villavicencio-García, V. Petranovskii, R. Vazquez-Duhalt, A. Huerta-Saquero

**Affiliations:** Centro de Nanociencias y Nanotecnología, Universidad Nacional Autónoma de México, Ensenada, Baja California, Mexico; Universidade de Aveiro, PORTUGAL

## Abstract

The antimicrobial activity of silver nanoparticles (AgNPs) is currently used as an alternative disinfectant with diverse applications, ranging from decontamination of aquatic environments to disinfection of medical devices and instrumentation. However, incorporation of AgNPs to the environment causes collateral damage that should be avoided. In this work, a novel Ag-based nanocomposite (CEOBACTER) was successfully synthetized. It showed excellent antimicrobial properties without the spread of AgNPs into the environment. The complete CEOBACTER antimicrobial characterization protocol is presented herein. It is straightforward and reproducible and could be considered for the systematic characterization of antimicrobial nanomaterials. CEOBACTER showed minimal bactericidal concentration of 3 μg/ml, bactericidal action time of 2 hours and re-use capacity of at least five times against *E*. *coli* cultures. The bactericidal mechanism is the release of Ag ions. CEOBACTER displays potent bactericidal properties, long lifetime, high stability and re-use capacity, and it does not dissolve in the solution. These characteristics point to its potential use as a bactericidal agent for decontamination of aqueous environments.

## Introduction

The unique physicochemical and antibacterial properties of silver nanoparticles (AgNPs) have placed them among the most widely commercialized nanomaterials in health care, e.g., for wound infection treatment and medical device disinfection, among others [1,2]. However, this antibacterial activity turns undesirable when AgNPs are discarded and dispersed; AgNPs end up dissolving and leaching ions that act against desirable microorganisms from aqueous or other environments [3,4]. The immobilization of AgNPs on an inert and nanostructured support as opposed to the traditionally used colloidal nanoparticles [5,6] shows important advantages because of the obtained chemical and thermal stability, which also prevents leaching [5,6]. In addition, AgNPs immobilization hinders nanoparticle oxidation, degradation and agglomeration, favouring long-term antimicrobial activity [7].

The antimicrobial activity of nanocomposites including Ag nanoparticles or ions has been amply documented. Among them, silica- and zeolite-AgNPs nanomaterials have been previously characterized [3,8] In a comparative analysis, Lalueza *et al*. [9] evaluated the bactericidal effect of several silver-based materials and revealed that bactericidal activity increases with silver content. It was also showed that biocidal effectiveness of silver-releasing materials increase concomitantly to the content of bioavailable ionic silver content [10].

On the other hand, zeolites have been considered a promising inorganic reservoir for hosting silver ions and to regulate their release. Thus, zeolites have become relevant in material sciences and bionanotechnology due to the potential to develop functional nanocomposites [11]. Zeolites provide a rigid, stable and biocompatible structure that is certainly useful for these purposes. In particular, zeolite-Ag nanocomposites have been suggested for antimicrobial applications due to their high ion exchange capacity, high surface area, supportive capacity of different types of nanoparticles, negative surface charge, chemical inertness and low or null toxicity [5,12,13]. These nanomaterials have been proposed for bactericidal applications such as disinfestation and treatment of water or surfaces [14].

For bactericidal applications, a complete characterization and evaluation of an antibacterial agent should be done. This includes clinically relevant parameters such as the minimum bactericidal concentration (MBC), the minimum inhibitory concentration (MIC), the median lethal dose (LD_50_), the bactericidal action time of the nanomaterial, the effective concentration and the time of exposure for a given bacterial load. Also, it is important to determine its re-use capacity and its bactericidal/bacteriostatic effect [2,5,15–19]. To this end, it is also necessary to establish a systematic study of the antimicrobial properties of the nanomaterial taking into account aspects related to its stability, management and recovery, in order to prevent environmental impact.

In this work, we present the synthesis and the structural and chemical characterization of a new Ag-based nanocomposite (labeled as CEOBACTER) constituted by nanoparticles and clusters of Ag grown and strongly bonded to mordenite-type zeolitic matrices. A complete protocol of its bactericidal activity, reporting a systematic study of all above mentioned parameters, is presented. Finally, through dialysis experiments, we demonstrate that the release of silver ions from CEOBACTER, is sufficient to exert a bactericidal effect, even without direct contact between AgNPs and bacteria.

## Materials and Methods

### Synthesis and characterization of the silver-mordenite nanocomposite

The CEOBACTER nanocomposite was synthesized by a one-pot, solvent-free and organic template-free variant of the sol-gel process. Briefly: CEOBACTER samples were synthesized by direct synthesis performed following the methodology described in patent application MX/a/2012/013218 [20]. This process involves 110 ml of a mixture of 0.1 M aqueous solutions of sodium silicate and aluminum sulfate (with SiO_2_/Al_2_O_3_ molar ratio of 15) that was stirred for 30 min; then, 30 ml of 0.1 M aqueous solution of AgNO_3_ was mixed with the first solution. The final solution, with pH value of 9 ± 1, was autoclaved at 155°C for 48 h. Thereafter, the mixture was thoroughly washed and then air-dried [21]. CEOBACTER powders thus obtained were analysed physico-chemically. Chemical composition, morphology and crystalline structure of CEOBACTER were studied using a combination of different techniques. The structural characterization was performed by X-ray diffraction (XRD) with a Philips X’Pert diffractometer (with CuKα radiation and step/acquisition time of 0.02°/ 1 s) and by scanning transmission electron microscopy (STEM) using a JEOL JEM-2010 (with accelerating voltage of 200 kV). Powders morphology was examined by scanning electron microscopy (SEM) using a JEOL JSM-5300 microscope. The morphology and distribution of nanoparticles was explored by STEM, through to high resolution (HRTEM), bright-field, and Z-contrast imaging modes. Global chemical analysis was performed by inductively coupled plasma-atomic emission spectroscopy (ICP-AES) using a Variant Liberty 110 Spectrometer. The electronic state of the Ag atoms in the nanoparticles was study with X-ray photoelectron spectroscopy (XPS) experiments done in a SPECS system equipped with a PHOIBOS WAL electron energy analyzer using a monochromatic Al anode. All data were acquired using Al Kα X-rays (1486.6 eV, 200 W), with a pass-energy of 50 eV, a 0.1 eV step size, and a high-intensity lens mode; moreover, the diameter of the analyzed area was 3 mm, the chamber pressure was kept lower than 1 x 10–8 mbar, and the accuracy of the binding energy (BE) values is ± 0.1 eV. The UV-Vis spectroscopy using an AvaSpec ULS2048-UA-50 spectrophotometer was used to evaluate the silver plasmonic activity. The physical adsorptive properties were examined by BET and Langmuir analysis by N2 adsorption using a Tristar II 3020 Surface Area Analyser.

### Strain, media and bacterial growth conditions

*Escherichia coli* strain MC4100 was cultured at 37°C in Luria-Bertani (LB) broth/agar media (broth: 1% tryptone, 1% sodium chloride, 0.5% yeast extract; agar plates: 2% bacteriological agar added, adjusted to pH 7.5). The culture media were prepared using distilled water and sterilized by conventional methods.

For all experimental assays, cells were grown overnight (14–16 h) on LB at 37°C and 180 rpm in an incubator (Thermo SCIENTIFIC MAXQ 6000 model 4353). Then cell density of the culture was adjusted to 0.1 OD at 600 nm using a spectrophotometer (ThermoFisher SCIENTIFIC Oy type 1510), followed by serial dilutions to obtain 1x10^4^ cells per mililiter (cells/ml), except for evaluation of the effect of CEOBACTER on different bacterial load.

### Antimicrobial properties of CEOBACTER

The MIC (minimum inhibitory concentration [22]) and the MBC (minimum bactericidal concentration [23]) were determined for *E*. *coli* by the following microdilution test: once the initial inoculum of 1x10^4^ cells/ml was prepared the cultures were exposed to different concentrations of CEOBACTER (1.0, 1.5, 2.0, 2.5, 3.0, 3.5, and 4.0 μg/ml) in 100 μl of LB (by quintuplicate), and then incubated overnight at 37°C and 180 rpm. Experiments were done in 96-well U bottom plates. After incubation, treated cells were measured by spectrophotometry (for MIC determination) at 600 nm with a MultiskanGO plate reader (Thermo). Then, for MBC determination, 10μl of the same cultures were inoculated into LB-agar plates and re-incubated overnight at 37°C.

The LD_50_ (the concentration of CEOBACTER able to inhibit bacterial growth by 50%, compared with untreated control) was determined for the *E*. *coli* initial inoculum of 1x10^4^ cells/ml exposed to MBC and lower concentrations of CEOBACTER for two hours at 37°C and 180 rpm; then, the lethal dose was estimated as described above for MBC determination.

To demonstrate inertness of sodium-mordenite (NaMOR) matrix on bacterial growth, different NaMOR concentrations without Ag nanoparticles (ranging from 65 to 260 μg/ml) were tested.

### Bactericidal/bacteriostatic effect of CEOBACTER

An important feature to be evaluated regarding the bactericidal behaviour of nanomaterials is to determine whether the agent is bacteriostatic or bactericidal. The bacteriostatic effect prevents bacterial growth, keeping the culture in a stationary phase of growth, while the bactericidal effect kills the bacteria [24]. The bacteriostatic or bactericidal effect of CEOBACTER was determined by re-inoculation assays. *E*. *coli* cultures (1x10^4^ cells/ml) were exposed overnight to MBC in LB liquid cultures. Then 100 μl of this culture were taken to inoculate 5 ml of fresh LB medium without nanocomposite. Afterwards, the cultures were incubated overnight (at 37°C and 180 rpm) and then, bacterial growth was evaluated measuring the optical density. Also, 10 μl of the same cultures were inoculated into LB-agar plates and re-incubated overnight at 37°C.

### Time of the CEOBACTER bactericidal action

To determine the action time of the Ag-nanocomposite as microbicide, 3 ml of cultures previously adjusted to 1x10^4^ cells/ml were exposed to MBC of CEOBACTER and then samples of 10 μl were taken every hour during 24 h. Afterwards, each sample was inoculated into LB-agar and incubated overnight at 37°C. The colony-forming units per millilitre (CFU/ml) were determined to evaluate bacterial growth. Experiments were done in triplicate.

### Silver concentration dependence of the bactericidal activity

To determine this, cultures adjusted at 1x10^4^ cells were exposed to 1X, 2X, 4X, 8X and 16X CEOBACTER MBC (corresponding to 3, 6, 12, 24, and 48 μg/ml, respectively). Next, samples of 10 μl were taken at 0, 10, 20, 40, 60, and 120 min. Samples were inoculated into LB-agar and incubated overnight at 37°C to evaluate the growth. Experiments were carried out in triplicate.

### CEOBACTER lifetime

Once the bactericidal action time was determined, the CEOBACTER re-use capacity of nanomaterial was evaluated. To this, cultures of 1x10^4^ cells/ml were exposed to 1X, 2X, and 3X CEOBACTER MBC (3, 6, and 9 μg/ml, respectively) and then, culture samples were taken every two hours (period of time required to eliminate 1x10^4^ bacteria exposed to MBC of CEOBACTER). Next, culture samples were re-inoculated with the same amount of bacteria (1x10^4^ cells). This procedure was done continuously during 20 h. Then, the samples were inoculated into LB-agar and incubated overnight at 37°C for CFU plate counting. All experiments were done in triplicate.

### CEOBACTER bactericidal effectiveness

To evaluate the CEOBACTER bactericidal efficiency, different bacterial inocula were incubated with CEOBACTER MBC (3 μg/ml). Briefly, cells were grown overnight in LB broth at 37°C and 180 rpm; then, fresh 2 ml culture samples were adjusted to different cell concentrations: 1x10^4^, 1x10^5^, 1x10^6^, 1x10^7^, 1x10^8^, 3.75x10^8^, 7x10^8^, 1.2x10^9^, and 1.8x10^9^ cells/ml. Cell density was measured by spectrophotometry at 600 nm based on McFarland index [25] and then adjusted at the above densities. Next, the cultures were exposed to MBC of CEOBACTER and incubated overnight at 37°C and 180 rpm. After the treatment, culture samples of 10 μl were inoculated into LB-agar plates and incubated overnight at 37°C. Experiments were performed in quintuplicate.

### Ion-mediated bactericidal effect of CEOBACTER

In order to evaluate whether the release of silver ions from CEOBACTER is sufficient to exert a bactericidal effect, without any contact between the AgNPs and bacteria, dialysis experiments were performed. Confined *E*. *coli* bacterial cultures into dialysis sacks (3 KDa pore exclusion, Slide-A-Lyzer, Thermo Scientific) were exposed to CEOBACTER in LB media. Dialysis membranes avoid direct contact of CEOBACTER and bacterial cells. Briefly, cells were grown overnight in LB broth at 37°C and 180 rpm; then, fresh 2 ml culture samples were adjusted to 1x10^4^ cells/ml and placed into dialysis sacks. Next, dialysis was performed in LB media containing 9 μg/ml of CEOBACTER. 5 μl of culture samples from dialysis sacks were taken at 0, 1, 2, 4 and 8 hours, inoculated into LB-agar plates and incubated overnight at 37°C for CFU plate counting. All experiments were done in triplicate.

On the other hand, to demonstrate that the nanoparticles cannot pass through the pores of the dialysis sacks, 2 ml LB without nanomaterial were introduced into dialysis sacks and exposed to equivalent concentrations of colloidal AgNPs and CEOBACTER (120μg/ml) in LB media. After 12 hours of dialysis, samples of LB medium inside the dialysis sacks and the surrounding medium were taken and analysed to determine the Ag ion concentration by ICP-AES, as well as the presence of AgNPs by UV-VIS spectra analysis.

## Results and Discussion

### Structural and chemical study of CEOBACTER

**Fig 1:**
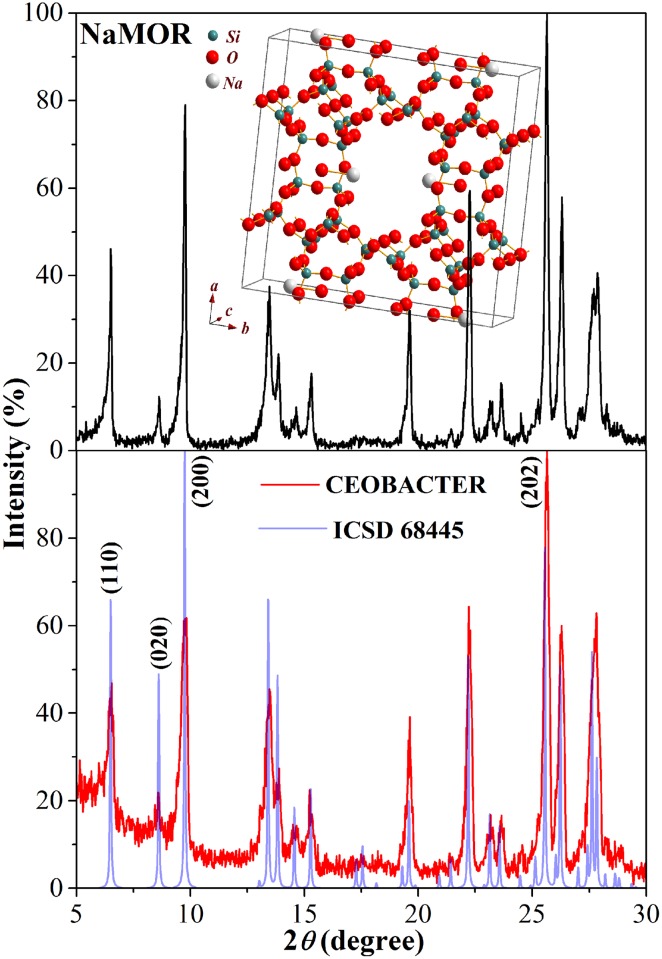
XRD analysis. XRD patterns of the CEOBACTER, the Na-mordenite (NaMOR) and the standard NaMOR ICSD 68445 file [26]. Inside, the NaMOR unit cell is shown.

The X-ray diffraction pattern of CEOBACTER random powders was compared with those of the synthesized Na-mordenite (NaMOR) without AgNPs and of the standard NaMOR reported in the 68445 file, from Inorganic Crystal Structure Database (ICSD) [26]. As can be seen in the Fig 1, CEOBACTER and NaMOR patterns are equivalent to that of ICSD 68445 file exhibiting good defined single phase mordenite-type zeolite structure with high crystallinity, where no reflections of the metallic silver are present as evidence of the absence of bulk-like particles; and not shift of the peak positions are observed as indicative that the mordenite lattice in the CEOBACTER is unaffected by the presence of Ag atoms, clusters and nanoparticles. However, for CEOBACTER and NaMOR samples, significant differences with the theoretical pattern may be identified; the most relevant is the decrease of the reflexion intensity and the observed left broadening of peaks corresponding with the (1 1 0), (0 2 0), and (2 0 0) planes, which intercept with the central line of the unit cell along to 12 members (12M) channel of the mordenite framework (inside of Fig 1), parallel to the (0 0 1) direction. Meanwhile, the CEOBACTER exhibits the (0 2 0) and (2 0 0) peaks with smaller intensity than those of the NaMOR sample.

**Fig 2:**
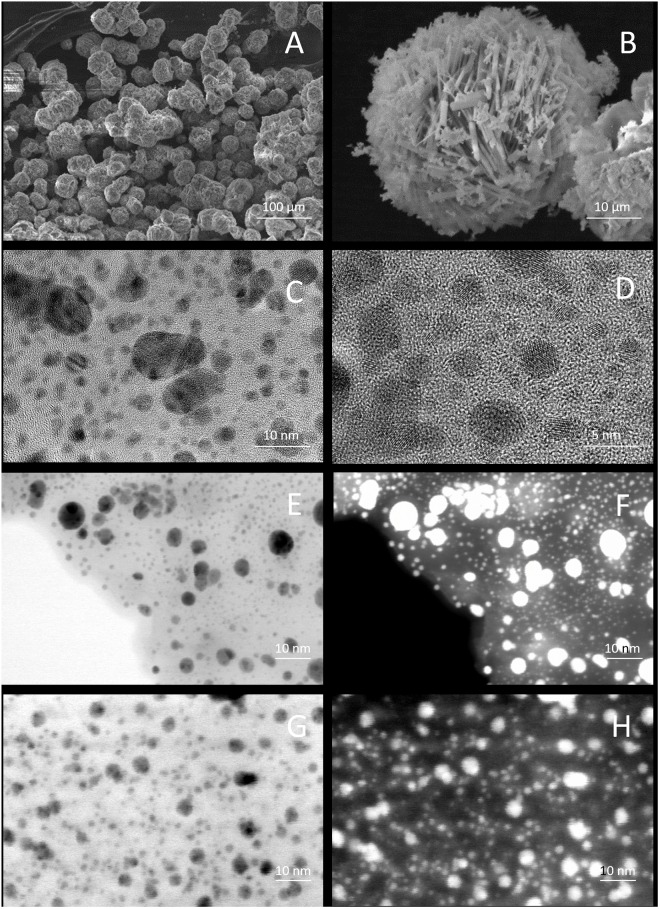
CEOBACTER characterization. SEM images of CEOBACTER powders (A) and needle-shape crystal grains (B). STEM micrographs in high resolution mode (C and D) showing the distribution, crystal planes, and orientations of AgNPs (dark zones) on the CEOBACTER nanocomposite. Micrographs in bright-field (E and G) and Z-contrast (F and H) modes before (E and F) and after (G and H) exposition to bacterial cultures.

Representative images from SEM and STEM analysis are illustrated in the Fig 2. As can be observed in the SEM image of Fig 2A, CEOBACTER powders samples exhibit spheroidal-shape grains with 40 μm average diameter. Such grains are formed by packing of needle-shaped crystals (Fig 2B), which is typical of mordenite crystals of low Si/Al ratio [27]. These needle-shaped crystallites, having 70 nm average diameter (obtained from TEM analysis for all synthesized samples), grow along the *c* crystallographic direction in good correspondence with previous reports [13,21,28]. Thus, such growth direction and the nanosize of crystallites diameter explains the decreasing and broadening of the first three XRD peaks for the CEOBACTER and NaMOR, with respect to those of the standard mordenite as was previously reported on similar composites [21,28,29]; whereas, the higher intensity decrease of such reflections in the CEOBACTER pattern can be assumed due to incorporation of the Ag atoms into the 12M channel along the *c* crystallographic direction which affect the diffraction from planes parallels to *c* axis. All this, justify that while the (2 0 0) reflection is the principal peak (100%) for the ICSD pattern, for the compounds synthesized in this work the main reflection corresponds to the (2 0 2) plane (the second most intense for ICSD pattern), which is used for normalize the CEOBACTER and NaMOR patterns. It worth to note that the (202) reflection should be affected by the incorporation of the Ag atoms inside the 12M channel, but as we do not have any reference to make a relative comparison, such effects on the peak are masked.

Moreover, from TEM imaging observations we did not find any vestiges of separated AgNPs. As can be seen in the Fig 2C and 2D, such AgNPs (dark zones) grow bonded homogeneously dispersed at the surface of the mordenite matrix, similar to previous reports on CdxZnySδOγ and Ag-CdxZnySδOγ nanoparticles supported on mordenite [13,21,28].

**Fig 3:**
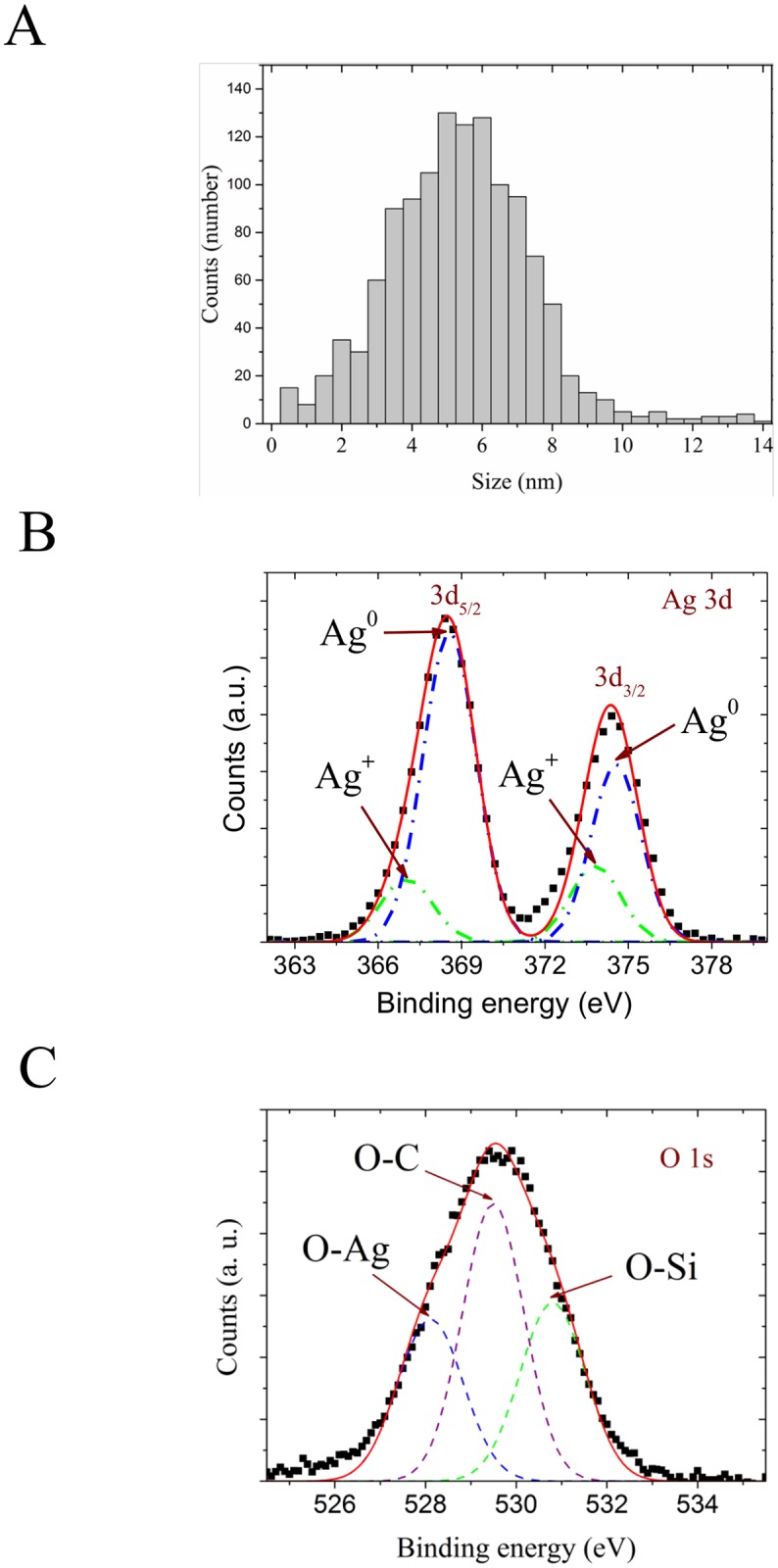
Histogram for the Ag nanoparticles size (A) and high-resolution Ag 3d (B) and O 1s (C) XPS spectra of the CEOBACTER. Solid line curve correspond to full multi-Gaussian fit, and dash-dot line curves show the deconvoluted signals.

In the meantime, from statistical analysis, the obtained size distribution in the Fig 3A shows an average size of AgNPs embedded in the mordenite matrix of 5 nm, with a narrow range in size from 1 to 10 nm. The size of the AgNPs is crucial for their bactericidal capacity, the smaller ones being the more effective since they offer a higher contact surface that facilitates the release of Ag ions [30].

The global chemical composition results of CEOBACTER powders obtained by ICP-AES are presented in Table 1, where values of the Si and Al atomic percentages, and the Si/Al ratio are in good correspondence with other reports of synthesized mordenite [13,28]; whereas the Ag content agree with the volume fraction occupied by AgNPs respect to total volume of the crystallites observed from TEM analysis.

**Table 1:** ICP-AES chemical and surface area analysis of CEOBACTER.

Atomic (%)				Surface area (m^2^/g)	
Si	Al	Ag	Si/Al	BET	Langmuir
28.1	4.3	1.5	6.5	167.2	254.8

Thus, to confirm the bonding of AgNPs on the zeolite surface XPS measurements were performed and the results for Ag 3d and O 1s spectra are illustrated in the Fig 3B and 3C, respectively. For silver spectrum (Fig 3B), the profile and the maximum position of the 3d_3/2_ and 3d_5/2_ peaks and the bonding energy difference of 6 eV between them, are in good agreement with other studies on AgNPs [31,32]. As can be seen, both peaks 3d_3/2_ and 3d_5/2_ show a noticeable left-asymmetry and thus, fitting process were used to deconvolute the Ag^+^ and Ag^0^ components. The higher peaks centred at 368.6 and 374.6 eV are attributed to metallic Ag^0^, while the lower peaks at 367.0 and 373.8 eV are assigned with the oxidized Ag^+^ [33]. From the oxygen spectrum (Fig 3C), where the peak exhibits both-side asymmetry, the fitting process shows three components centred at 528.4, 530.1 and 531.2 eV, which are associated with oxygen core-level signals coming from Ag-O bonds, carbonated species, and the Si-O bonds of the zeolitic matrix, respectively [34]. These results are in good correspondence with the previous characterization, allowing us to declare that the AgNPs are bonded to the surface of the zeolitic matrix.

On the other hand, the obtained surface area values, particularly those associated with the microporosity from the BET and the mesoporosity from the Langmuir methods, are relatively high in spite of the presence of AgNPs. This feature indicates a great contact surface which is significant for the interaction of CEOBACTER with the surrounding environment.

### Bactericidal properties of CEOBACTER

The MIC, MBC, and LD_50_ values obtained for *E*. *coli* were determined in LB liquid cultures and are reported in Table 2. As shown, the MBC and MIC concentrations of CEOBACTER are lower than those obtained for colloidal AgNPs [35] and as low as those reported for a zeolite-silver nitrate composite also tested in *E*. *coli* [36]. When comparing CEOBACTER with Zeo-AgNPs (faujasite zeolites doped with AgNPs, [15]) we found a better bactericidal efficiency (MIC of 2 vs 19.4 μg/ml), even its lower silver content (15 vs 23 mg/g). This difference may be due to the bacterial inhibition method used, since agar dilution method used in that report is more qualitative than quantitative. Antimicrobial inhibitory concentrations found for CEOBACTER suggest greater efficiency, compared with those for free AgNPs, which may be attributed to a homogeneous distribution of the Ag nanoparticles in the zeolitic matrix [37,38]. In this regard, agglomeration of free nanoparticles seems to reduce their effectiveness, a feature that is overcome by obtaining new nanomaterials with homogeneous nanoparticle distribution, such as CEOBACTER (Fig 2C and 2D). As a control, the parent NaMOR matrix was separately assayed to determine whether bactericidal properties of CEOBACTER only originate from silver nanoparticles. No bacterial growth inhibition was observed, even at a concentration as high as 260 μg/ml of NaMOR (data not shown).

**Table 2:** Bactericidal parameters (expressed as μg/ml) of the CEOBACTER nanocomposite compared with those of Zeolite-Ag nanocomposites and colloidal AgNPs.

	MBC (μg/ml)	MIC (μg/ml)	LD_50_ (μg/ml)	AgNPs size (nm)	Ag-loading content (mg/g)	Reference
**CEOBACTER**	3	2	1.6	5(±3)	15	This work
**Zeo-Ag**^**+**^	3.5	1	ND	ND	365.7	[36]
**Zeo-AgNPs**	ND	19.4	ND	ND	23	[15]
**AgNPs**	50–60	ND	ND	12.3(±4.2)	-	[39]
**PVP-AgNPs**	ND	10	5.5	17.9(±7)	-	[35]

ND: Not determined

Regarding the bactericidal or bacteriostatic activity of the nanocomposite, after exposing *E*. *coli* cultures to MBC of CEOBACTER overnight, the re-inoculation of fresh medium did not restore bacterial growth. These results confirm that the CEOBACTER effect is bactericidal rather than bacteriostatic, feature that is important in clinical practice [40].

### Time of bactericidal action of CEOBACTER

**Fig 4:**
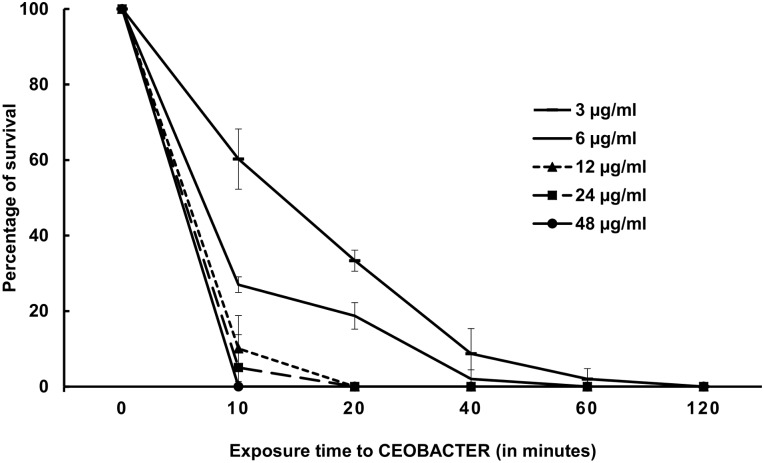
Exposure time and agent concentration dependence of bactericidal effect of CEOBACTER. The values are the average of three different experiments.

Before bactericide agents are used with disinfectant purposes, the minimum exposure time and concentration required for total bactericidal effect must be estimated. As shown in Fig 4, CEOBACTER eliminated bacterial growth in two hours at a concentration of 3 μg/ml. To determine if a concentration increase reduced the time required to kill all bacteria, 1X, 2X, 4X, 8X, and 16X of MBC were tested over *E*. *coli* cultures (1x10^4^ cells/ml). Fig 4 shows that increasing CEOBACTER concentration drastically reduces the time required to obtain the bactericidal effect. Thus, doubling the CEOBACTER concentration (2 times MBC) reduces the time needed to eliminate the bacterial load, from 120 minutes to 40 minutes; if it is doubled again from 12 and 24 μg/ml, the bacterial load is eliminated in only 20 minutes. For 48 μg/ml only 10 minutes are required.

### CEOBACTER operational lifetime

Bactericide re-use is an increasingly important commercial parameter [41]. We determined the number of times that CEOBACTER could be re-used without losing its bactericidal properties. Table 3 shows the number of inocula (and number of bacteria) that were eliminated by the same amount of nanocomposite, following the experimental procedure described above. As shown in Table 3, using 1X MBC (3 μg/ml), bacterial survival reached 98% after the sixth bacterial load compared with the control; however, for 2X MBC (6 μg/ml) 12% bacterial survival was observed after six re-inoculations, and 100% survival was reached in the eighth re-inoculum. Finally, for 3X MBC (9 μg/ml), 12% bacterial survival was observed after the ninth re-inoculum and 100% at the eleventh re-inoculum.

**Table 3:** Bacterial survival values (in percentage) for different CEOBACTER concentrations at increasing number of inoculums.

Number of inoculums	[CEOBACTER] μg/ml		
	3	6	9
**1**	0%	0%	0%
**2**	0%	0%	0%
**3**	0%	0%	0%
**4**	5% (±0.4)	0%	0%
**5**	55% (±4.1)	0%	0%
**6**	98% (±12.4)	12% (±7.5)	0%
**7**	100%	86% (±6.5)	0%
**8**	100%	100%	0%
**9**	100%	100%	12% (±4.1)
**10**	100%	100%	98% (±13.7)
**11**	100%	100%	100%

The percentage of bacterial growth was determined in comparison with bacterial growth without CEOBACTER, taken as 100%. Standard deviations are shown in parenthesis. Each inoculum was 1 x10^4^ cells/ml.

These effective results of CEOBACTER re-use may be associated with the fact, as evidence showed, that the AgNPs are strongly bonded to the mordenite matrix which releases Ag ions depending on the surrounding chemical environment. In this regard, TEM analysis of CEOBACTER after exposure to bacterial cultures, showed that the nanomaterial is not physically altered, and AgNPs are not released to the medium (Fig 2G and 2H). The CEOBACTER operational lifetime may be limited by the following factors: *i*. The bacterial load may exceed the amount of Ag ions that can be released by CEOBACTER; and/or *ii*. The possibility that molecular components of dead bacteria sequester the ions released by CEOBACTER.

### CEOBACTER bactericidal effectiveness

**Fig 5:**
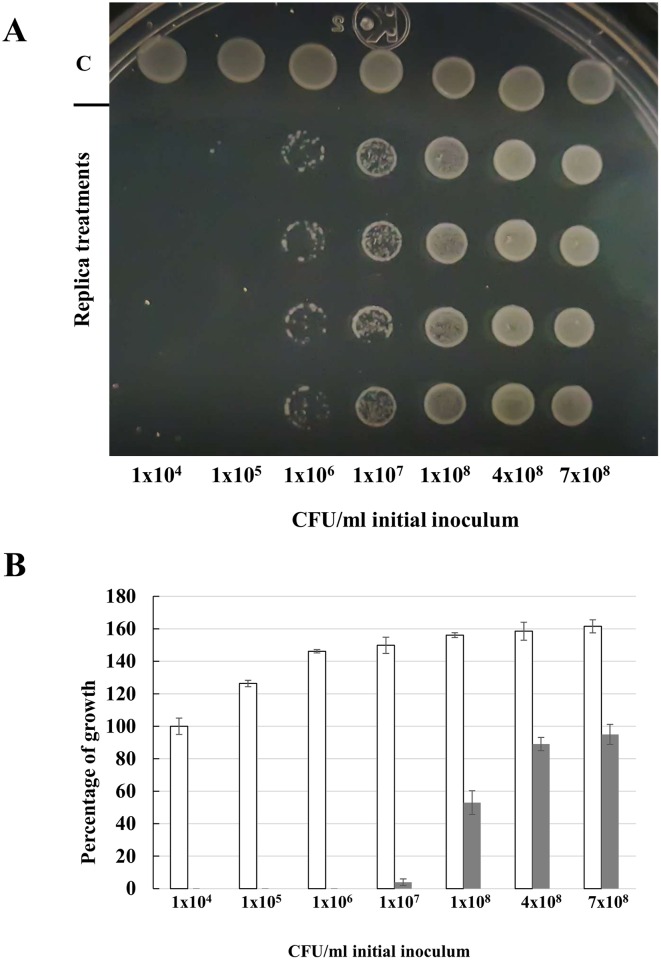
CEOBACTER bactericidal effectiveness. Different bacterial concentrations were incubated with MBC of CEOBACTER (3 μg/ml) during 2 h to test viability. A) Control (C) and four replica treatments (columns) for each initial inoculum are shown. In B) Percentage of bacterial growth at different initial inoculum. Bacterial growth at 1x10^4^ initial inoculum without CEOBACTER was taken as 100%. Bacterial growth without CEOBACTER (white bars) and bacterial growth exposed to CEOBACTER (black bars) are shown.

The effect of the bacterial load on the bactericidal capacity of CEOBACTER was determined. As shown in Fig 5, when the number of inoculated bacteria is increased the CEOBACTER bactericidal capacity may be exceeded. The CEOBACTER MBC (3 μg/ml) showed a partial inhibitory effect up to 1x10^7^ of initial inoculum, whereas for a bacterial load of 1x10^8^ or higher its bactericidal capacity was reduced to negligible (Fig 5A). However, when analysing bacterial growth by optical density at 600 nm, taking into account each initial inoculum in the absence and presence of CEOBACTER (Fig 5B), it was found that even at high concentrations of initial inoculum, growth inhibition is still observed, although this is not enough to completely eliminate the bacterial load.

However, we tested higher CEOBACTER concentrations and found that for an initial inoculum of 1x10^8^ minimal bactericidal CEOBACTER concentration was of 9 μg/ml and for 7x10^8^ bacteria of 15 μg/ml. Using the right amount of nanomaterial for a given bacterial load allows controlled application and avoids excessive use of nanomaterial, which could generate its accumulation.

### Ion-mediated bactericidal effect of CEOBACTER

**Fig 6:**
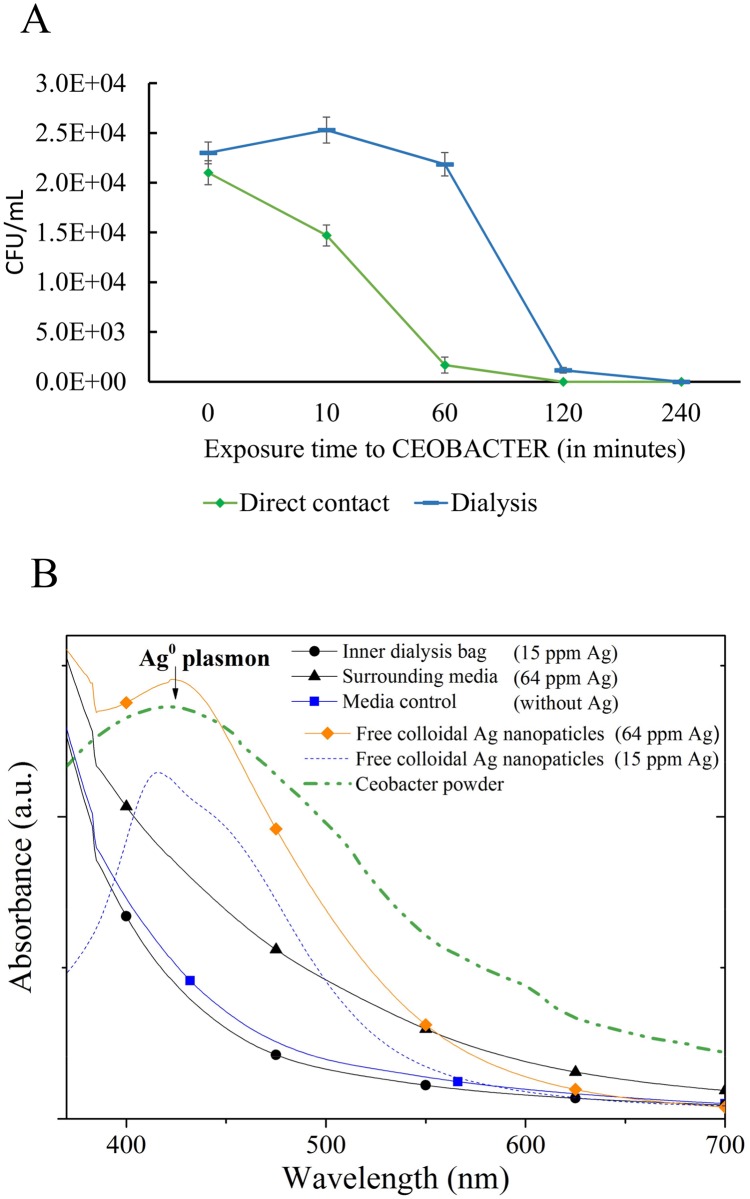
AgNPs-ion release is sufficient to exert a bactericidal effect over *E*. *coli* cultures. By dialysis experiments, confined bacteria was killed by ions released from CEOBACTER (A). No release of AgNPs were shown in culture media inside and outside dialysis sacks (B). Ag ions was measured by ICP analysis. Free colloidal AgNPs at reported concentrations were used as a sensitivity control.

Controversy exists regarding the possible mechanism of action of silver nanoparticles on microorganisms [8,18,35,42–44]. In particular, if the bactericidal effect of silver nanoparticles is due to direct contact of the nanomaterial with bacteria, or by the release of ions which alter the membrane and cause cell death, or by a combination of both effects. In this regard, we performed a series of experiments to demonstrate that silver ions release is sufficient to exert antibacterial activity, without any contact between the silver nanoparticles and bacteria (Fig 6). By dialysis experiments, confined bacterial cultures were exposed to CEOBACTER in LB media. Dialysis membranes avoid direct contact of CEOBACTER and bacterial cells. However, the bactericidal capacity of CEOBACTER remained, although it showed a delay (Fig 6A). On the other hand, the delay in the effective time of antibacterial activity of CEOBACTER on confined bacteria in comparison with that found in direct contact CEOBACTER-bacterial cells experiments, suggests that direct contact between CEOBACTER and bacteria can have an antibacterial effect as well, or that the time for Ag ions diffusion to achieve a steady-state concentration into dialysis sacks takes longer and therefore the time required for the antimicrobial effect is also longer (Fig 6A). This result is in agreement to those obtained by Hsueh, et al., 2015 for *Bacillus subtilis*, showing that AgNPs exert microbial toxicity through the release of Ag^+^ ions that subsequently penetrate into bacterial cells and are oxidized intracellularly to Ag^2^O [43]. On the other hand, ICP analysis of media samples inside and outside dialysis sacks, resulted in the presence of 15 ppm of Ag ions inside and 64 ppm of Ag ions outside, respectively (Fig 6B, insert). This result strongly suggests that Ag ions release from CEOBACTER migrate into dialysis sacks and this is sufficient to kill bacteria. Additionally, we conducted UV-VIS absorption spectrum analysis with the aim of finding metallic silver inside and outside the dialysis sacks. The absorbance spectra of the samples showed no release of metal nanoparticles to the medium during bacterial growth inhibition assays (Fig 6B). As a control, the UV-VIS absorption spectrum of CEOBACTER powder shows a peak near above to 400 nm corresponding to the surface plasmon resonance characteristic of metallic AgNPs in good agreement with other report of colloidal AgNPs with size lower than 40 nm [29].

The use of a nanocomposite such as CEOBACTER as antimicrobial agent has outstanding advantages. The characterization of CEOBACTER showed that the silver nanoparticles were homogeneously dispersed and attached to the zeolitic matrix, which prevents the release of the nanoparticles. Additionally, the nanocomposite showed high thermal stability and resistance to pH changes [21]. These unique properties make this nanocomposite suitable for use in decontamination processes. The synthesis of CEOBACTER is an easy, scalable and inexpensive procedure compared to other nanomaterials in which nanoparticles are functionalized in some matrices (composed of silica, polymers, etc.) that are more expensive [8]. It should be noted that these nanomaterials, as in our case for CEOBACTER, have shown higher antimicrobial activity compared with free AgNPs, and similar to the activity obtained using silver ions. This higher antimicrobial efficiency is attributed to the stability conferred by the matrix in which AgNPs are embedded and also to its homogeneous distribution along the surface, which enables continuous release of silver ions and avoids nanoparticle agglomeration.

The use of nanoparticles in the biomedical or environmental fields raises concerns about the safety of their use, especially in humans. Recently, Zhang et al. (2014) reported an excellent evaluation of the potential toxicity of nanoparticles in biological systems [45]. Our novel antimicrobial nanocomposite CEOBACTER does not release nanoparticles, minimizing the public health risk. Nevertheless, all new nanomaterials require previous systematic study of their physical and antimicrobial properties and further studies are needed to assess their toxicity and set security levels for use.

## Conclusions

CEOBACTER, a nanocomposite based on AgNPs embedded on zeolite matrix, was successfully grown by a one-pot, template-, solvent-, and seed-free route. Nanocomposite characterization revealed growth of silver nanoparticles over the volumetric surface of the zeolitic matrix, with a narrow size distribution. The structure of the matrix constrained further agglomeration by self-assembly of the nanoparticles, which showed strong attachment to the mordenite surface. The novel nanocomposite exhibited high antibacterial activity, stronger than commercial products based on colloidal AgNPs. The CEOBACTER nanocomposite has advantages over free AgNPs since it avoids dispersion of detrimental silver nanoparticles into the environment, and allows recovery and re-use of the material.

Among the parameters left to evaluate, we propose the determination of MBC, MIC and LD_50_ for different microorganisms, and the assessment of the nanocomposite’s microbicidal action time, its operational lifetime and specific concentration needed to remove increasing bacterial loads. It is also important to establish the possibility of inactivation and/or recovery of the nanocomposites for final disposal. On the whole, the bactericidal properties, stability and re-use capacity of CEOBACTER point to it as a promising bactericidal agent for decontamination of aqueous environments and even for clinical applications.
